# The mouse cortical meninges are the site of immune responses to many different pathogens, and are accessible to intravital imaging

**DOI:** 10.1016/j.ymeth.2017.03.020

**Published:** 2017-08-15

**Authors:** Jonathan A. Coles, Phillip J. Stewart-Hutchinson, Elmarie Myburgh, James M. Brewer

**Affiliations:** a Centre for Immunobiology, Institute of Infection, Immunity and Inflammation, College of Medical, Veterinary and Life Sciences, University of Glasgow, Glasgow, United Kingdom; b Department of Pediatrics, Division of Infectious Diseases, Washington University School of Medicine, St. Louis, MO 63110, USA; c Centre for Immunology and Infection, Department of Biology, University of York, York, United Kingdom

**Keywords:** Multiphoton, Intravital, Pathogen, Immune response, Dura mater, Leptomeninx

## Abstract

A wide range of viral and microbial infections are known to cause meningitis, and there is evidence that the meninges are the gateway to pathogenic invasion of the brain parenchyma. Hence observation of these regions has wide application to understanding host-pathogen interactions. Interactions between pathogens and cells of the immune response can be modified by changes in their environment, such as suppression of the flow of blood and lymph, and, particularly in the case of the meninges, with their unsupported membranes, invasive dissection can alter the tissue architecture. For these reasons, intravital imaging through the unperforated skull is the method of choice. We give a protocol for a simple method of two-photon microscopy through the thinned cortical skull of the anesthetized mouse to enable real-time imaging with sub-micron resolution through the meninges and into the superficial brain parenchyma. In reporter mice in which selected cell types express fluorescent proteins, imaging after infection with fluorescent pathogens (lymphocytic choriomeningitis virus, *Trypanosoma brucei* or *Plasmodium berghei*) has shown strong recruitment to the cortical meninges of immune cells, including neutrophils, T cells, and putative dendritic cells and macrophages. Without special labeling, the boundaries between the dura mater, the leptomeninx, and the parenchyma are not directly visualized in intravital two-photon microscopy, but other landmarks and characteristics, which we illustrate, allow the researcher to identify the compartment being imaged. While most infectious meningitides are localized mainly in the dura mater, others involve recruitment of immune cells to the leptomeninx.

## Highlights

• Many pathogens cause inflammation of the brain meninges.
• Two-photon microscopy can video cell movements in the meninges of reporter mice.
• Immune cells are recruited mainly to either the dura mater or the leptomeninx.
• Guidance to distinguishing dura, leptomeninx, and parenchyma *in vivo* is given.

## Graphical abstract

The objective of a two-photon microscope is shown above the thinned parietal skull of a mouse. Infection with *Plasmodium berghei* (left hand side, blue spheres) causes recruitment of T cells (orange with grey nuclei) to subarachnoid spaces in the leptomeninx. Infection with *Trypanosoma brucei* (right hand side, blue) causes recruitment of T cells to the dura where they can move among the collagen fibers (green lines). A near-infrared laser beam (large arrow) excites fluorophores by two-photon absorption and they emit fluorescence at characteristic visible wavelengths (colored arrows).

**Figure:**
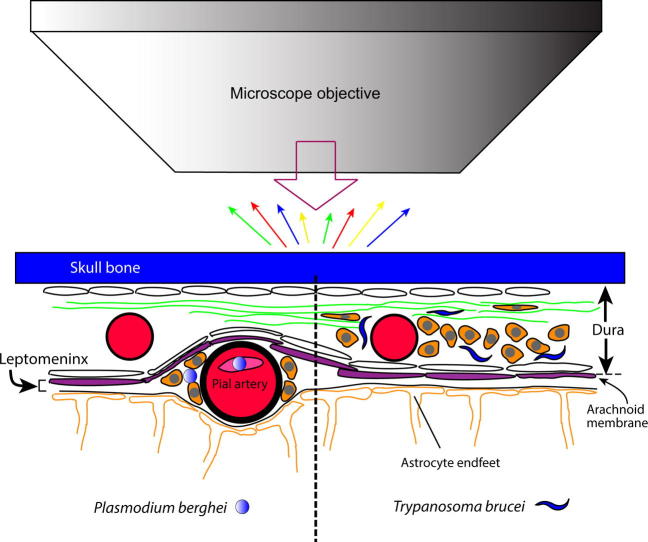

## 1 Investigating immune responses in the cranial meninges

The very wide range of pathogens that can cause meningitis includes viruses [1], [2], [3], [4], bacteria [5], [6], [7], protozoa [8], [9], [10], [11], nematodes [12], and fungi (notably *Cryptococcus* [13], [14]). A common symptom in humans is headache, which implies nervous activity in the meninges, and activation, at the very least, of resident mast cells [12], [15], [16]. However, knowledge of meningeal immune responses to microbial infection is meagre, so the great convenience of working with mouse models, notably the availability of reporter mice in which selected classes of cells express fluorescent proteins (e.g., [17], [18]), outweighs the disadvantage that pathological changes in the transcriptome may not perfectly mimic those in humans [19], [20]. To date, the few reports of immune responses to pathogens in compartments of the cranial meninges have been based on studies on mice; these have described recruitment of neutrophils [21], and T lymphocytes and monocytes [10], [11], [21].

In vivo imaging, by recording movements and interactions of pathogens and different classes of immune cells, can give information, not only of numbers and location, but also about the activation states of immune cells. User-friendly programs, such as Volocity or Imaris, can extract the position co-ordinates of cells at each time-point in a video, draw the tracks, and calculate the speed and the x,y,z, components of the velocity of each cell for each time interval, or the angle turned between two time intervals [22], [23]. From these values, statistics can be calculated such as the mean speed along a track [21], the displacement rate (the rate at which a cell moves further from its starting point), the mean x,y and z components of velocity [10], the fraction of time intervals during which the cell is stationary (the ‘arrest coefficient’ [11], [21]), the direction of movement relative to blood vessels [24] or the duration of interactions between two cells [25]. These parameters can change markedly during infection.

The architecture of the meninges, with its large spaces separated by flimsy membranes, is difficult to preserve if the overlying skull is removed, and an important part of an immune response is extravasation of cells (or pathogens) from flowing blood. It is therefore desirable to observe meningeal pathophysiology in vivo and through the skull. The most informative technique to date is two-photon microscopy, which is based on the theoretical prediction of Maria Göppert-Mayer [26] [27]. Perhaps surprisingly, the skull bone overlying the cortical meninges is an asset for imaging, rather than a handicap. When an area of the bone is mechanically thinned, it provides a transparent window to the meninges, while the surrounding bone provides a solid anchorage for immobilizing the imaging field. Other laboratories have described methods for making a window that remains transparent over weeks or months and allows repeated imaging sessions [28], [29], the video by Marker et al. [30] being particularly recommended. Our priorities are slightly different. We have chosen not to allow mice to recover from anesthesia, for the scientific reason that the stress of the imaging session would modify the disease progression, for the practical reasons that a quick and reliable preparation is convenient and it can be interesting to image other parts of the mouse after dissection, and for the ethical reason that disease progression should be terminated as soon as clinical symptoms develop. Statistically significant data on the movements of immune cells require videos lasting several tens of minutes at several sites per mouse, with an accurately controlled temperature. We have therefore chosen a fairly quick, non-sterile preparation, adjustable gas anesthesia, and perfusion of the chamber over the thinned area. A perfusion system had initially been set up for ex-vivo experiments [25], [31]. It is useful during thinning (for removing debris and for cooling), and, while the mouse is under the two-photon microscope, to control the temperature above the thinned skull and to maintain the water contact with the objective during video imaging lasting tens of minutes, and for subsequent ex vivo imaging at a controlled temperature.

### 1.1 The functional anatomy of the meninges

Popular views of the structure of the cortical meninges have been influenced by text-book images based on an artistically very attractive drawing published by Weed in 1923 [32]. This shows the dura as a thin membrane overlying a capacious subarachnoid space (SAS) in the leptomeninx. While this is true over the basal brain, over the convexities of the cortex, as Weed [33] himself stated, “the subarachnoid space is only of capillary thickness”. In primates, the SAS extends into sulci, which are bridged by the dura, but mice and other rodents have no sulci, and over the dorsal brain the SAS is deep only where is penetrates the medial longitudinal fissure. In contrast, the dura is thick enough to accommodate bundles of collagen, a rich vasculature, and extracellular lacunae [34], [35]. It is extensively innervated [36] and activity in trigeminal neurons with endings in the dura is perceived as headache (see [37]). The dura also contains lymph vessels, which were described in man by Mascagni in 1787 (reproduced in [38]), and have been shown in rat [36] and in mouse [39], [40].

The functional compartmentation of the meninges is still not clear. Electron microscopy shows that the cells of the outer layer of the leptomeninx, the arachnoid membrane, are connected by tight junctions [41] and it can be assumed that it is this membrane that separates the cerebrospinal fluid (CSF) in the SAS from the extracellular fluid of the dura [42]. The nature of the barrier, as well as its location, is of interest as there is some evidence that particulate matter and T-cells can pass from the leptomeninx into lymph vessels within the dura [40], [43] and, in the opposite direction, T-cells may pass from the dura to the perivascular spaces of vessels penetrating the parenchyma [10], [44]. The CSF present in the SAS can contain pro-inflammatory cytokines secreted by the choroid plexus [45], [46] so the paths of flow of CSF are relevant to the immunopathology of the meninges and the brain parenchyma. There is agreement that CSF reaches the dorsal SAS via channels adjacent to cerebral arteries (e.g. [43], [47]) but uncertainty about whether or not there is flow of CSF down the paravascular spaces of arteries penetrating the parenchyma, and up the paravascular spaces of emerging veins [37], [48], [49], [50], [51], [52].

**Fig. 1:**
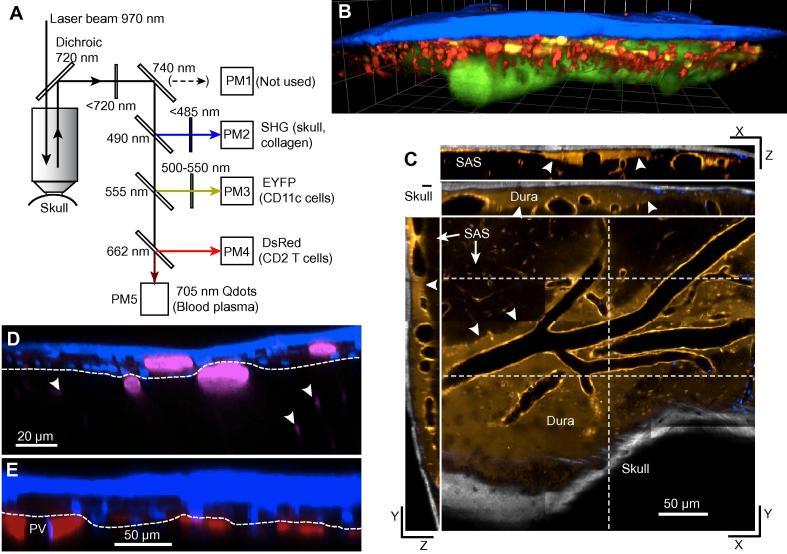
A,B. Imaging several fluorophores. A. An example of an arrangement of dichroic mirrors and filters that was used for imaging second harmonic generation (SHG), EYFP, DsRed and 705 nm quantum dots. The dichroics and filters shown were chosen from those available and are not necessarily optimal. PM4 and PM5 are GaAsP photomultiplier tubes, which have higher sensitivity than the multi-alkali photomultipliers PM1-3 at long wavelengths; for this reason the long wavelength signal was directed to PM5 rather than PM1. B. 3D reconstruction of a four-color Z-stack obtained with the optics in (A). The mouse had been infected with *Trypanosoma brucei* 27 days earlier. T cells (red) and CD11c+ cells (yellow) are seen close under the skull (blue). Emission from the quantum dots in the blood plasma is shown as green. C. Planes from a Z-stack in a ‘DsRed’ mouse expressing DsRed under control of a *β* actin promoter and cytomegalovirus enhancer cassette [53]. DsRed is shown as bronze and SHG from the skull as grey. An XY plane, slightly oblique to the plane of the meninges, is shown, including skull (lower right) and leptomeninx (upper left) and also one YZ plane and two XZ planes that cut the XY plane at the white dashed lines. There is labeling of vascular walls and, apparently, of the dural tissue. Arrowheads point to what we suggest is the arachnoid membrane. D. A vertical section showing (in blue) skull by SHG and nuclei labeled by i.v. injection of furamidine, and (in pink) blood plasma labeled with quantum dots. Three faintly visible intraparenchymal vessels are indicated by arrowheads. We suggest that the dashed white line follows the boundary between dura and leptomeninx. Excitation at 824 nm. E. A vertical section 59 min after infusion in the cisterna magna of Texas Red, which labels extracellular channels in the leptomeninx. Blue indicates the skull (by SHG) and nuclei labeled by i.v. injection of furamidine. We suggest that the dashed white line follows the boundary between dura and leptomeninx. Excitation at 810 nm. (For interpretation of the references to colour in this figure legend, the reader is referred to the web version of this article.)

In vivo two-photon microscopy, which allows simultaneous imaging of several fluorophores (Fig.1A), in Z-stacks that can be reconstructed as 3D images (Fig.1B), is expected to contribute to resolving these questions. Here, we focus on whether a given immune response is in the dura or in the leptomeninx.

### 1.2 Locating cells and structures in the mouse meninges

**Fig. 2:**
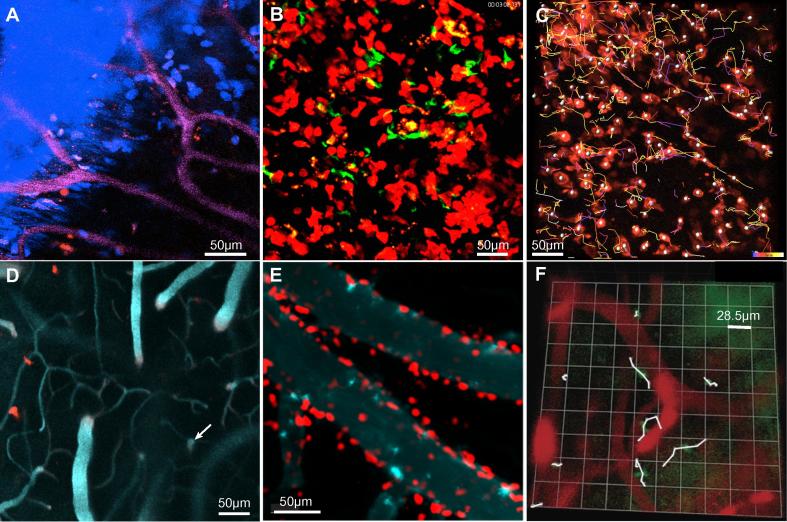
In vivo images of T cells in hCD2^+^ DsRed mouse cranial meninges seen through the thinned skull. A-C show mainly the dura; D-F mainly the leptomeninx. A. Dural blood vessels (magenta), skull bone and collagen (blue, by SHG), dural cell nuclei (blue, labeled by i.v. injection of furamidine), two or three CD2^+^ T cells expressing DsRed. Small red spots are of unknown origin. B. The dura of a mouse infected 11 days earlier by i.p. injection of *Trypanosoma brucei*. T cells express DsRed, trypanosomes GFP. (A,B from [10].) C. Tracks of T-cells from a video lasting 21 min. 27 days post infection with *T. brucei*. The excitation was at 1116 nm (using an OPO beam - see Section 2.1.1). D. Large horizontal pial vessels lie in depressions in the cortical surface then plunge abruptly into the parenchyma where characteristically sinuous horizontal capillaries are seen. Examination of vertical reconstructions shows that the two DsRed T cells are in the leptomeninx. Blood plasma labeled with 705 nm Qdots. E. In a mouse infected 5 days earlier with *Plasmodium berghei*, T cells are recruited to spaces adjacent to leptomeningeal blood vessels. (From [11].) F. In a mouse with permanent occlusion of the middle cerebral artery, those T cells (faint GFP expression) that move are seen to remain close to blood leptomeningeal vessels. (From [55].) (For interpretation of the references to colour in this figure legend, the reader is referred to the web version of this article.)

Blood vessels can be labeled by i.v. injection of fluorescently labeled dextran, or inert quantum dots (see Section 2.2. for more details); in Fig.1B they are shown in green. Bone, and also collagen, are readily imaged with a two-photon microscope using the phenomenon of second harmonic generation (SHG, [54]). A very intense pulse of light from a near-infrared laser generates the second harmonic, so that, for example, an exciting wavelength of 900 nm produces emission at 450 nm, which can be used to construct an image as if from a conventional fluorophore (blue in Fig.1B). Blood vessels in the dura are relatively leaky, and fluorescent nuclear dyes injected i.v. rapidly label nuclei in the dura, as well as nuclei of vascular endothelial cells throughout the mouse [9], [10]. This is seen in the vertical sections shown in Fig.1D,E and also in Fig.2A. In Fig.1D we suggest where the arachnoid membrane might be located, below the labeled nuclei and above leptomeningeal vessels. This interpretation is supported by images obtained after infusion in the cisterna magna of dye which flows via the basal meninges to reach the cortical leptomeninx where it fills channels that define at least part of the subarachnoid spaces (Fig.1E, [10], [43], [48]). Shaw et al. [11] imaged ‘DsRed’ mice that express DsRed under control of a *β* actin promoter and cytomegalovirus enhancer cassette [53]. As shown in Fig.1C, in these mice, not only are the vessel walls labeled, but the tissue of the dura, but not the leptomeninx, is weakly fluorescent. This may turn out to be a useful tool for distinguishing the two compartments, but a reporter mouse in which the arachnoid membrane is fluorescent would be more convincing.

While the mouse is under the microscope, a quick, provisional identification of the compartment being imaged can be made from XY images. Features of the dura include the proximity of the skull, and the presence of collagen and of nuclei labeled with an i.v. dye (Fig.2A). Capillaries are not present in the leptomeninx, while the small vessels in the dura have a characteristic branching pattern, seen in Fig.2A and beautifully illustrated by Key and Retsius (1875 [34]). The brain parenchyma is readily identified by the absence of large horizontal vessels and the characteristic sinuosity of the horizontal capillaries (left side of Fig.2D). The leptomeningeal vessels are large and horizontal and often lie in depressions in the parenchymal surface. Arteries plunge abruptly into the parenchyma, and veins emerge (Fig.2D). If necessary, arteries can be distinguished from veins by following their courses (veins mainly drain medially to the superior sagittal sinus) or by using reporter mice [48].

### 1.3 Immune responses in the dura and in the leptomeninx

Within two weeks of intraperitoneal infection, *Trypanosoma brucei* extravasates into spaces close under the skull. The trypanosomes can be seen squeezing among collagen fibers, and moving close to small blood vessels, but above most of the large ones, and they move mainly within volumes about 50 µm across [9], [10]. These features indicate that they are in the dura. Their arrival is preceded by invasion by T cells. T cells and trypanosomes coexist in the same planes (Fig.2B), and the T cells appear to move in spaces unrelated to blood vessels (Fig.2C), suggesting that they also are in the dura. CD11c+ cells are also recruited to this space (Fig.1B). However, late in infection, occasional T cells are seen below the dura, at the level of the pia [10]. It is not known whether this meningeal inflammation contributes to invasion by trypanosomes of the brain parenchyma, which may occur by extravasation from capillaries [56], [57] or via CSF [58], [59].

A different pattern is seen with infection by *Plasmodium berghei*. Recruited T cells tend to lie along large vessels (Fig.2E, [11]), presumably in the leptomeninx, and their movement tends to be parallel to the vessel. This is also seen after occlusion of the middle cerebral artery (Fig.2F [55]).

The situation is less clear in infection by lymphocytic choriomeningitis virus. In a pioneering work, Kim et al. [21] imaged infected mice through the thinned skull. CD8^+^ cells specific for the virus (P14 cytotoxic T lymphocytes [CTLs]) and LysM^+^ myelomonocytic cells were recruited to the meninges. The CTLs appear to induce recruitment of myelomonocytic cells, which then compromise vascular integrity and initiate fatal convulsive seizures. The CTLs lie mainly in a plane separated by a space from the skull (their Fig. 1A), and LysM^+^ cells appear to be mainly in the dura at day 5, but at day 6 they appear throughout the thickness of the meninges and extend below the pia (their Figs. 4p,q). The virus itself was found in cells in widely distributed sites through the meninges including fibroblasts (their Fig. S4c) and cells overlying the glia limitans (their Fig. S4c). It seems possible that use of ‘DsRed’ mice (Fig.1C) [53] could allow unambiguous identification of the locations of the LysM+ cells. Further information on immune cells, in at least the outer layers of the dura (see [35]) has been obtained by fluorescence activated cell sorting (FACS) and by gene expression analysis, using the pooled tissue obtained by scraping five calvaria [15].

## 2 Methods

### 2.1 Apparatus

#### 2.1.1 Choice of microscope

The two-photon microscope should be upright with a motorized stage and focus. The objective should be corrected for water immersion, have good focusing in the near-infrared, high transmission in the visible, and a working distance >1 mm. The maximum spatial resolution of a laser-scanning microscope depends almost entirely on how well the scanning beam is focused in the tissue and this is determined mainly by the numerical aperture (NA) of the objective, and the wavelength (λ), as given by Abbé’s formula: resolution in the XY plane = λ/(2NA). There is further loss caused by scattering within the tissue and by lens aberration. Typical resolution in the Z direction is illustrated in Fig.1B,C,D,E. To achieve the maximum resolution, large numbers of photons per pixel must be detected, either because the object is strongly fluorescent, or because the object is stationary and scanned for a long time. (The uncertainty in the intensity of a pixel is √n, where n is the number of photons detected. For example, for an uncertainty <1%, >10^4^ photons must be detected.) An objective with low magnification is better than one with high magnification because it has a greater field of view, but maintaining a high NA makes it bulky and expensive [60]. The standard compromise is a ×20 objective with NA ≈ 1. The speed of scanning can be increased by zooming, and this is useful for videoing e.g. the rapid extensions and retractions of the dendrites of a dendritic cell. A ×10 objective can be useful for finding regions of interest.

The excitation source is a titanium-sapphire femtosecond laser. An adjustable wavelength is a great advantage. The addition of an optical parametric oscillator (OPO), which converts some of the energy of the incoming (‘pump’) beam to a longer wavelength (typically 1000–1200 nm) gives two further advantages: it allows optimal excitation of some red-emitting fluorophores, and if part of the original pump beam is combined with the OPO beam, a bigger range of signal sources can be imaged simultaneously. The imaging program should allow automatic intensity adjustment during acquisition of a Z-stack (so that the image of the deepest XY plane is as bright as in the topmost XY plane). For imaging lymphocytes, a scan rate of 8–10 frames/s is normally adequate; for imaging very motile cells, such as trypanosomes, faster acquisition, achieved by zooming, could be useful. Blood velocity [61] and vessel pulsations [49] can be measured by line scanning, and do not require a specially high scan rate. It is useful to have at least four detectors for different spectral bands so that, e.g., blood, skull bone and collagen (by SHG), T-cells, monocytes, and parasites can be imaged simultaneously (Fig.1A).

#### 2.1.2 An example of a microscope system

We use a Zeiss LSM7 MP microscope with excitation light from a Ti-sapphire femtosecond laser tunable from 700 to 1050 nm (Chameleon Ultra II, Coherent, Santa Clara, USA). The output of the Ti-S laser passes through an optical parametric oscillator (OPO, Coherent): when pumped by the Ti-S laser at about 800 nm, outputs up to 1200 nm are obtained. It is possible to use part of the pump beam simultaneously with the OPO output. The intensities of the Ti-S beam bypassing the OPO, and the OPO beam are regulated by acousto-optical modulators controlled by the imaging program (Zen 2010, Zeiss). Almost all imaging is done with a 20× water immersion objective, NA 1.0. (W Plan-Apochromat, Zeiss). Excitation and emitted light are separated in the microscope nose by a dichroic mirror with a cutoff at 740 nm. Five detectors of non-descanned fluorescence are available, three multialkali, and two GaAsP photomultipliers (the latter having higher sensitivity at the longer visible wavelengths). The motorized stage is model H1P1A/LSM/B (Prior, Rockland, MA). The mouse plate need not be clamped to the stage, but must sit stably on it by being supported at its corners: a thin blob of epoxy glue at each corner may be necessary. The microscope is shielded from light by curtains hung from a frame made of RS Pro Aluminium Alloy Strut Profile (RS Components).

#### 2.1.3 Apparatus for skull thinning

**Fig. 3:**
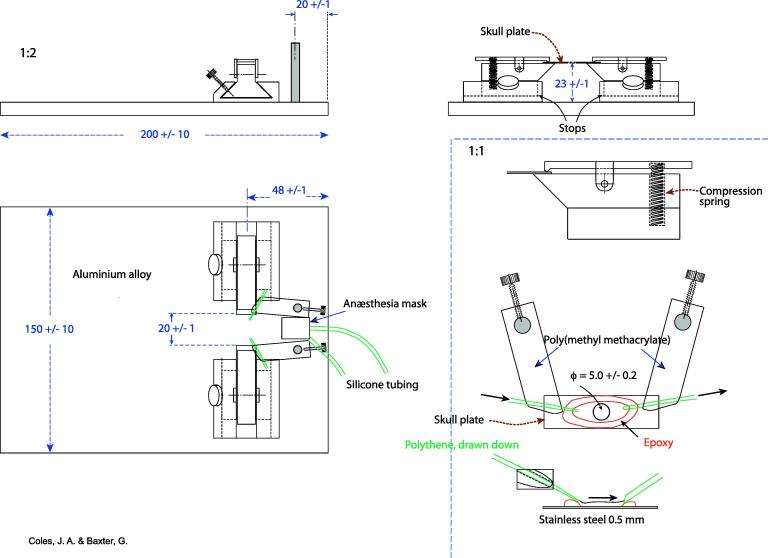
A system for holding the mouse skull by a ‘skull plate’ (bottom right) which is held by spring-loaded clamps on dovetails. The poly(methyl methacrylate) anesthesia mask is held over the mouse’s snout by the elasticity of the silicon tubes that deliver and extract the isofluorane anesthetic. For superfusion of the thinned skull with buffered saline, delivery and aspiration tubes are held on swivelling poly(methyl methacrylate) holders. The end of the aspiration tube should be cut at a bevel so that it sucks both air and liquid. The heating mat (not shown) is held by tape on the baseplate.
(4) A ‘mouse plate’: a base with fixtures for holding the skull plate (Fig. 3).
(5) A system for superfusing the skull (Fig. 4).

(1) A good quality dental drill with speeds to 30,000 r.p.m. with a spherical diamond burr < 1.5 mm in diameter (Diama International, London, UK).
(2) A dissecting microscope on a swinging arm, with illumination. Zoom is useful.
(3) A ‘skull plate’: a thin plate of stainless steel with a hole (bottom right in Fig. 3). A perfusion chamber is formed by a dyke of epoxy glue such as Araldite. With use, the expoxy loses its hydrophobicity: this can be restored with a PAP pen as used on slides for immunostaining, or, if necessary, with grease.

**Fig. 4:**
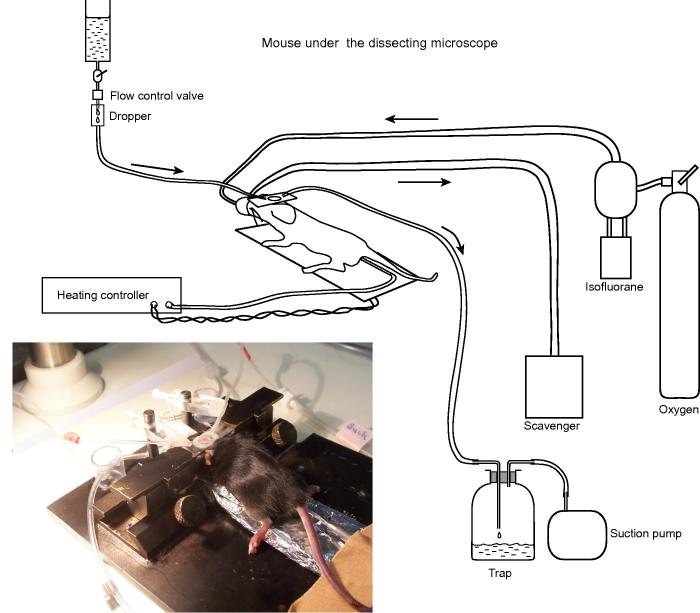
The arrangement for temperature maintenance, anesthesia and superfusion during thinning of the skull under a dissecting micrscope.
(6) A small suction pump (e.g. an aquarium pump). Since the noise of grinding is an invaluable feedback for thinning the skull, it is important that the suction pump be quiet and that there is little other noise.
(7) A homeostatic heating system controlled by a rectal thermistor probe. We use a TC-344B dual heating control by Harvard Apparatus with rectal probe 50-7221f and a heating mat made to order by De-Icers (MHG) Ltd, Cheltenham, UK. The heating mat should have a maximum power of about 6 W so with a 12 V supply the mat element should have a resistance of about 25 Ohm.
(8) An inline heater for superfusion of the skull while imaging (Fig. 4). E.g. 64– 0102, Warner, and controller. Unless a thermistor is included in the circuit close to the mouse, it is necessary to do a dummy experiment to find the temperature of the heater that maintains the fluid temperature at the mouse skull at 33–36 °C. (With this temperature range above the skull, T cells in the dura move at the same speeds as at 37 °C [10].
(9) A high quality dental cement such as RelyX Unicem Clicker, 3 M ESPE, Seefeld, Germany. (A cyanoacrylate glue such as ‘Vetbond’ is not as good).
(10) 200 ml of a physiological saline: NaCl 150 mM, KCl 2.5 mM, CaCl_2_ 2.0 mM, HEPES, hemisodium salt, 10 mM. We avoided phosphate buffer because phosphate chelates calcium, which is necessary for blood clotting. Use of the hemisodium salt of HEPES automatically makes the pH of the solution close to 7.5.
(11) Phosphate buffered saline (PBS) for intravenous injections.
(12) Stock solutions for i.v. injection of blood marker. E.g. dextran 70 kD labeled with fluorescein or rhodamine at 100 mg/(mL phosphate-buffered saline), or commercial suspensions of inert quantum dots (e.g., Qdots, Thermo Fisher). Furamidine can be useful for labeling nuclei of host cells and trypanosomes in the dura [9]. We made a stock solution of 1 mg in 40 µL DMSO.
(13) Dissecting tools. Scissors for cutting hair and scalp, a scalpel for cleaning the skull, small forceps for swabbing.
(14) An insulin syringe for injecting in the tail vein.
(15) Small metal ruler (50–100 mm long) for measuring coordinates on the skull.
(16) Fine-tipped black fiber tip pen to mark where the skull plate is to be centered.
(17) Surgical adhesive tape, such as Leukoplast, BSN medical, Luxembourg.
(18) Sharpened matchsticks, for mixing and applying glue.
(19) A test slide with a fluorescent object (typically a section of a *Lilium* stalk), to check that the imaging system is working.

#### 2.1.4 Anesthesia

A volatile anesthetic has the advantage that depth of anesthesia can be adjusted easily when the mouse is under the microscope. In the case of trypanosome infection we found that induction appeared to be stressful and to minimize stress we first anesthetize the mouse with a low dose of ketamine + medetomidine (10 mL/kg body weight, i.p.) and then place the mouse on the mouse plate equipped with an anesthetic mask. The mask is held in place by the elasticity of the silicone tubing of the delivery and scavenge tubes (Fig. 4). 1.5–2% isofluorane in oxygen suppresses the reflex response to a foot pinch without perturbing respiration.

*Important point:* Make sure that the mouse plate can be moved from the dissecting microscope to the two-photon microscope with all the tubing and wires connected (anesthesia, heat control, superfusion). The superfusion is briefly disconnected so that the inline heater can be inserted.

### 2.2 Planning the experiment

Spectra of two-photon absorption by many fluorescent proteins, and the resulting emission, are listed in [62], [63]. The absorption spectra have two peaks, one at long wavelengths, and one at shorter wavelengths, typically 900–1100 nm and <800 nm. Although it is usually the long wavelength that is useful, in some cases the short wavelength can be effective. Other two-photon absorption spectra are at http://www.drbio.cornell.edu/cross_sections.html. Emission spectra are available on many sites, including Thermofisher. Tests can be made in vitro, e.g., on a blood sample from a reporter mouse.

The fluorophores of reporter mice will be the starting point for choosing the excitation wavelength(s) and dichroic filters. CD2-DsRed (T-cells) [18], [64] and CDllc-EYFP (dendritic cells and CD11c+ monocytes [17]) can be simultaneously imaged with excitation at about 960 nm (Fig.1B). Blood can then be labeled with a marker with longer emission, such as 705 nm quantum dots (e.g., Qdots from ThermoFisher). SHG from skull bone and collagen will be produced at exactly half the excitation wavelength, in this case 480 nm. If two populations of cells are labeled with fluorophores with extensive overlap of the emission spectra, such as YGP and GFP, algebraic programs that subtract one channels from another will be necessary to track cells of each population separately. Other useful reporter mice include LysozymeM-GFP (myelomonocytes including neutrophils and macrophages, [21]) CX3CR1-GFP (microglia and some dendritic cells and macrophages, [65], [66]) and GFAP-GFP (astrocytes, [67]).

Prepare a skeleton protocol sheet on which details of the experiment will be noted. This will include: type of mouse, date post infection, dichroics and filters, fluorophores…., for each image file a unique identifier with space to note: Time, excitation wavelength(s), beam power, detector gains, image area, scan duration, depth of Z-stack, XYZ co-ordinates… Some of this information is stored by the computer with the image file, but it is convenient to have all the information in one place. We find a paper document to be more convenient and durable than a computer file. A basic Word file can be conveniently updated for each session (see example in S1).

### 2.3 Experimental procedure

**Fig. 5:**
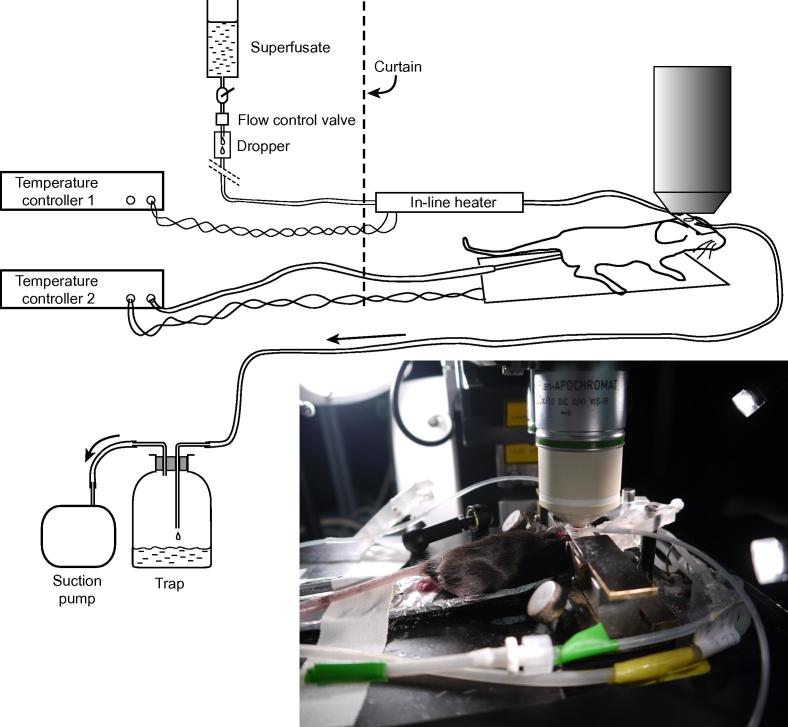
The mouse under the microscope. The superfusate is heated in an in-line heater. The anesthetic mask and its tubing are shown in the photograph, but not the diagram.
(18) After the imaging (up to 2–3 h), the mouse is euthanized by an overdose of anesthetic.

(1) Fill the reservoir of the skull superfusion system with the buffered saline and check that it flows (it is sometimes necessary to suck with a syringe).
(2) Insert the desired dichroics and filters in the detection beam. See Fig.1A for an example.
(3) Switch on the laser and microscope and use a test slide (typically a section of a *Lilium* stalk) to check that the imaging is functioning. Set the laser wavelength. Switch on the anesthetic scavenge pump and the suction pump.
(4) Anesthetize the mouse by i.p. injection or by isofluorane in an induction box. Place it on the heating mat on the mouse plate and turn on the oxygen flow (about 0.3 L/min) through the isofluorane, set at 1.5–2%. Insert the greased rectal probe and switch on the heat controller output. *Important point:* Take care never to switch on the heating if the rectal probe is not inserted. Unless there is feedback from the probe, the heating mat can overheat.
(5) Check that the mouse does not react to pinching a foot and that the breathing is normal. If necessary, adjust the anesthetic concentration.
(6) Using scissors, thin the hair over the dorsal calvaria. Section and retract the scalp over the left parietal cortex. Wipe away the periosteum using a cotton bud, and clean the bone surface with a scalpel over an area about 10 mm across.
(7) Choose a convenient point on the skull as the center of the imaging field. This is usually about 2 mm lateral and 2 mm posterior to bregma, unless the aim is to be close to the superior sagittal sinus (for example, to image lymph vessels). Mark the point with the fiber-tip pen and measure (and write down) the coordinates. Place the skull plate on the skull with the pen mark in the center of the hole and check that there is enough exposed bone to allow the plate to sit snugly. Wipe the skull plate.
(8) Prepare dental cement. The volume can be rather less than the quantity delivered by a full ‘click’ of the RelyX Unicem Clicker. The two components are mixed for 30 s with a sharpened matchstick.
(9) Using the matchstick, spread a thin ring of cement round the underside of the hole in the skull plate. Place the skull plate crossways on the skull with the pen mark near the center of the hole. Hold the plate firmly in place for 1 min. and check that it stays in place when released. (The cement will not yet be hard.) Note the time.
(10) Prepare the blood marker solution in an Eppendorf tube. For labeled dextran, add 50–70 µL stock solution, for Qdots, 20–30 µL of the commercial suspension, for furamidine, sufficient DMSO stock solution for a final concentration of 10 mg/kg body weight. Add PBS to a total volume of 100 µL and draw the mixture into the insulin syringe.
(11) Warm the tail of the mouse in a large Petri dish of hot tap water, or by holding it against the heat mat, and inject the blood marker in a tail vein.
(12) Seven min. after gluing the skull plate, clamp it to the supports on the mouse plate. Check that the plate is well-sealed on the skull. Small leaks can be filled with more cement. If the plate is not well-glued to the skull, it is necessary to clean the plate and skull and start again.
(13) Keep checking the mouse breathing, and core temperature, which should be 36.5–37.5 °C.
(14) Position the inflow and outflow tubes for the superfusion and start the flow at about 1 drop per 3 s (Fig. 3, Fig. 4).
(15) Zoom the dissecting microscope until the hole in the skull plate half fills the field. Set the dental drill speed to 30,000 r.p.m. and, supporting it with both hands, clear off surplus cement and start thinning the skull. Clear the debris either by flushing with an increased superfusion rate or with a scrap of laboratory tissue.
(16) Increase the zoom so that the hole in the skull plate fills the field. Moving the burr over the surface so that the noise of grinding can just be heard, continue thinning until the meningeal vessels, including small ones, are visible. There may be temporary bleeding from diploic and emissary vessels, but usually from no more than one or two points in the field. Stop the perfusion inflow and drain the thinned area using the outflow sucker. The relief of the surface becomes visible and areas needing further thinning can be identified. Reflood the field and make finishing touches, moving the burr along the horizontal projection of the drill axis. Thinning takes 10–15 min.
(17) Move the mouse plate, with all associated tubing and wires, to the microscope stage. Stop the superfusion, insert the inline heater (Fig. 5) and restart the superfusion. Raise the stage until the objective lens is immersed in the superfusate. Focus on the surface of the skull using oblique illumination from a fiber light guide, or on meningeal blood vessels using the epifluorescence mode of the microscope. Lower the curtains round the microscope. Set the detectors at high gain and the laser power at minimum. Switch on the laser beam and increase the power until an image is visible. *Important point:* always set the excitation power low before opening the laser beam shutter.

*Time:* The total procedure, from induction of anesthesia to imaging the first video, takes up to 90 min.

## 3 Some outstanding questions

There is a huge amount to be learnt about if, when, and where, pathogens arrive in the meninges, the characteristics of the immune cells that precede or follow them, and the cytokines or other signals that co-ordinate the pathogenesis (see, e.g. [21]). In addition there are still major questions concerning the physiology of the meninges. The outer layers of the arachnoid are rich in mitochondria, and two possibilities are that metabolic energy is required to manage the boundary between the SAS and the dura [68] or (we suggest) that the mitochondria are part of a temperature control system (see [69]). Some observations suggest that T cells can move across the arachnoid membrane [40] but the mechanisms are unknown. The way amyloid beta is removed from the brain is debated, but there is agreement that part of the efflux passes through the meninges [51], [70]. As so often happens, pathologists pose questions for physiologists.

## Funding

This work was funded by the Bill and Melinda Gates Foundation [OPPGH5337] (http://www.gatesfoundation.org/), MRC (http://www.mrc.ac.uk/) award G0900487, and National Institutes of Health (http://www.nih.gov) grants R01 AI055037 and T-32-CA009161. The Wellcome Centre for Molecular Parasitology is supported by core funding from the Wellcome Trust [085349] (http://www.wellcome.ac.uk/). The Zeiss 710 MP microscope at the Skirball Institute of Biomolecular Medicine was available through the Microscopy Core via NCRR (http://www.nih.gov/about/almanac/organization/NCRR) S10 grant RR023704-01A1.
