# Supplementary material for: The mouse cortical meninges are the site of immune responses to many different pathogens, and are accessible to intravital imaging
Source: Methods. 2017 Aug 15;127:53–61. doi: 10.1016/j.ymeth.2017.03.020 (PMC5595162; doi:10.1016/j.ymeth.2017.03.020)
Supplement: Supplementary data 1 [file mmc1.docx]

**171031A CD2 DsRed x CD11c 10 dpi *T. b. brucei*.**

Mouse: Infected 171021 Age 10 wks Wt Sex

PPL: Susan Smith Parasitaemia:

**Aims:**

**1. Verify that there are no tryps below the pia.**

**2. Fast time series for counting tryps in dura.**

**3. Z-stacks for counting TCs and CD11c+ cells.**

**4. Extravasation of plasma. LFTS** ['Long Fast Time Series']

**5. Deep ZSTS** ['Z-stack Time Series'] **for TCs (for tracking)**

**6. Cardiac perfusion, freeze brain.**

Main dichroic 740 nm, output filter 720 nm SP,

Dichroics: D1 662 (DsRed) ; D2 490 + 485 SP; D3 empty; D4 555 (FITC/ DsRed)

Anaesthesia, ketamine +medetomidine 0.1 mL/10 g Isofluorane. Coordinates: Lat Post Bleeding?

3M RelyX Unicem Time:

655nm QDs 30 uL, furamidine 10 μL, PBS 70 uL. Time:

Are tryps ever present in perivascular space? Find an artery. ZS, ZSTS, ZS.

171031A_01 Time = Lambda = nm Power = G2 =

G3 = G4 = G5 = Zoom = Size Scan time = Z = z = N =

Total time= Coordinates: z = x = y =

171031A_02 Time = Lambda = nm Power = G2 =

G3 = G4 = G5 = Zoom = Size Scan time = Z = z = N = Cycles = Interval =

Total time= Coordinates: z = x = y =

171031A_03 Time = Lambda = nm Power = G2 =

G3 = G4 = G5 = Zoom = Size Scan time = Z = z = N = Cycles = Interval =

Total time= Coordinates: z = x = y =
